# Development of Thermostable Lyophilized Sabin Inactivated Poliovirus Vaccine

**DOI:** 10.1128/mBio.02287-18

**Published:** 2018-11-27

**Authors:** Woo-Jin Shin, Daiki Hara, Francisca Gbormittah, Hana Chang, Byeong S. Chang, Jae U. Jung

**Affiliations:** a Department of Molecular Microbiology and Immunology, Keck School of Medicine, University of Southern California, Los Angeles, California, USA; b Integrity Bio Inc., Camarillo, California, USA; Icahn School of Medicine at Mount Sinai; University of North Carolina-Chapel Hill; University of North Carolina-Chapel Hill

**Keywords:** Sabin inactivated poliovirus vaccine, cold chain, thermostable, lyophilization, D-antigen

## Abstract

As oral poliovirus vaccine (OPV) causes vaccine-associated paralytic poliomyelitis, the polio endgame strategy introduced by the Global Polio Eradication Initiative calls for a phased withdrawal of OPV and an introduction of inactivated poliovirus vaccine (IPV). The introduction of IPV creates challenges in maintaining the cold chain for vaccine storage and distribution. Recent advances in lyophilization have helped in finding a temperature-stable formulation for multiple vaccines; however, poliovirus vaccines have yet to capture a stable, safe formula for lyophilization. In addition, efficient *in vitro* methods for antigen measurement are needed for screening stable vaccine formulations. Here, we report size exclusion high-performance liquid chromatography (SE-HPLC) as a reliable means to identify the leading lyophilized formulation to generate thermostable Sabin inactivated poliovirus vaccine (sIPV). High-throughput screening and SE-HPLC determined the leading formulation, resulting in 95% D-antigen recovery and low residual moisture content of sIPV following lyophilization. Furthermore, the lyophilized sIPV remained stable after 4 weeks of incubation at ambient temperature and induced strong neutralizing antibodies and full protection of poliovirus receptor transgenic mice against the *in vivo* challenge of wild-type poliovirus. Overall, this report describes a novel means for the high-throughput evaluation of sIPV antigenicity and a thermostable lyophilized sIPV with *in vivo* vaccine potency.

## Abstract (precis)

Poliomyelitis is a highly contagious disease caused by the poliovirus. While the live attenuated OPV has been the vaccine of choice, a major concern is its ability to revert to a form that can cause paralysis, so-called vaccine-associated paralytic poliomyelitis. Therefore, the new endgame strategy of the Global Polio Eradication Initiative includes the introduction of an IPV. However, the feasibility of the use of current IPV formulations in developing countries is limited, because IPV is insufficiently stable to be purified, transported, and stored under unrefrigerated conditions. We successfully designed the sIPV for use in the dry state that maintains the full vaccine potency in animal models after incubation at ambient temperature. This report provides, for the first time, candidate formulations of sIPV that are stable at elevated temperatures.

## INTRODUCTION

Poliovirus (PV) is a member of the Picornaviridae family in the order of Picornavirales and a causative agent of poliomyelitis. PV is formed in nonenveloped capsid and has a single-stranded positive-sense RNA genome (1, 2). The three serotypes of poliovirus, PV1, PV2, and PV3, have slightly different capsid proteins that define cellular receptor specificity and virus antigenicity. PV1 is the most common form encountered in nature and is highly localized to regions in Pakistan and Afghanistan. PV2 was declared eradicated in September 2015 after last being detected in October 1999 in Uttar Pradesh, India, and PV3 has not been seen since its detection in parts of Nigeria and Pakistan in 2012. All PVs can be transmitted person to person via direct contact, contaminated food, or other fomites. Poliovirus infection is asymptomatic or mild in about 95% of infected individuals, and approximately 0.5% of those may present paralytic disease. However, due to its highly contagious nature, poliovirus infection can affect large populations.

Vaccines are the most effective tool for controlling viral infection (3), evidenced by the eradication of smallpox virus (4) and the substantial reduction in the number of PV, Japanese encephalitis virus, influenza virus, and human papillomavirus infections (5). Two types of PV vaccines are currently used: an inactivated PV (inactivated PV vaccine [IPV]) given by injection that was developed by Jonas Salk in 1955 and a live attenuated PV (oral PV vaccine [OPV]) given by mouth that was developed by Albert Sabin in 1961 (6, 7). OPV proved to be superior in administration, eliminating the need for sterile syringes and making the vaccine more suitable for mass vaccination campaigns. OPV also provided longer-lasting community immunity than IPV (8). However, a potential (although rare) adverse effect of the OPV is its ability to revert to a pathogenic form that causes vaccine-associated paralytic poliomyelitis (VAPP) (9). Furthermore, outbreaks of VAPP caused by a circulating vaccine-derived PV have been reported (10). In 2017, the World Health Organization (WHO) reported 96 cases of VAPP in Syria and Democratic Republic of Congo. The Global Polio Eradication Initiative has played a major role in reducing the cases of poliomyelitis from 350,000 cases annually to 22 cases in 2017 (http://polioeradication.org/polio-today/polio-now/this-week). In 2016, the WHO successfully replaced trivalent oral poliovirus vaccine with bivalent oral poliovirus vaccine to eliminate the chance of generating type 2 circulating vaccine-derived poliovirus (11). Thus, the eradication of type 2 PV, the absence of detection of type 3 PV worldwide, and restriction of type 1 PV to only a few geographic areas of three countries has enabled the implementation of the endgame of polio eradication, which calls for a phased withdrawal of OPV and an introduction of IPV.

The introduction of IPV creates challenges in maintaining the cold chain for vaccine storage and distribution. This temperature sensitivity of IPV remains a significant hurdle during immunization campaigns (12). IPV can be stored for up to 4 years at optimal temperatures (2°C to 8°C); temperatures outside this range drastically reduce vaccine potency (13). Thus, there have been major efforts to improve the stability of IPV and to eliminate the need for the cold-chain process during distribution and storage, such as the use of an artificial hydrated silica exterior on virions (14), IPV encapsulation using microspheres (15), IPV delivery using biodegradable mini-implant Bioneedles (16), and IPV lyophilization (17, 18).

Attempts to lyophilize IPV have resulted in low recovery following lyophilization and poor stability at ambient temperatures (17, 19–21). While sufficient optimization of a lyophilized vaccine can substantially improve thermostability (21), it can become a cumbersome process without an efficient and effective *in vitro* method to evaluate vaccine potency. The potency of IPV determined by the *in vitro* assay is expressed in arbitrarily defined D-antigen units (D-AgU). The D-AgU was established in the early 1960s (22) following characterization of purified virus preparations by sucrose gradient centrifugation where two bands were identified. One, the D fraction (D-antigen), was associated with infectious virus with intact structure as revealed by electron microscopy and RNA content. The other, the C fraction (C-antigen), contained low infectivity with little RNA and possessed a structure that was similar to the structure of the heat-treated virus. As induction of neutralizing antibodies is associated with the immunization of intact virus structures (D-antigen) but not with the immunization of C-antigen viral preparations, the potency of IPV has been a function of the D-antigen content. Thus, efficient *in vitro* methods for D-antigen measurement are needed for screening stable vaccine formulations.

In this study, various surfactant-based formulations were screened for Sabin inactivated poliovirus vaccine (sIPV) lyophilization, and size exclusion high-performance liquid chromatography (SE-HPLC) (23) was implemented as a novel high-throughput formulation assay for D-antigen quantitation of sIPV. Finally, a room-temperature-stable sIPV prepared by leading formulation induced strong neutralizing antibodies and full protection against wild-type (WT) poliovirus challenge *in vivo*. This sIPV formular will not only facilitate the distribution of the vaccine without the need of refrigeration but also contribute to the poliovirus endgame introduced by the Global Polio Eradication Initiative.

## RESULTS

### sIPV preparation.

**FIG 1:**
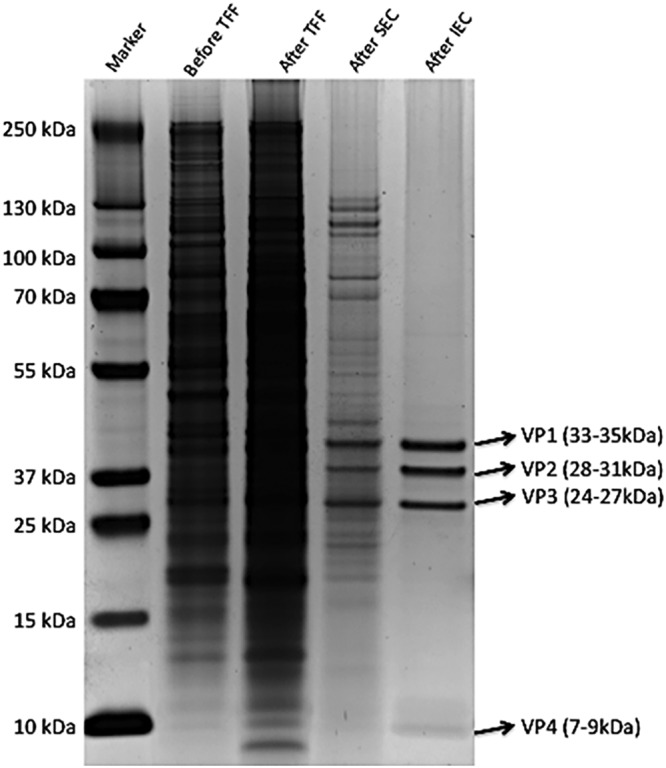
Purified inactivated Sabin poliovirus. Polioviral particles are comprised of icosahedral capsid proteins that consist of VP1, VP2, VP3, and VP4. To check the purity of Sabin inactivated poliovirus, virions were separated using SDS-PAGE followed by silver staining. Lane 1, molecular weight standards; lane 2, before tangential flow filtration (TFF); lane 3, after TFF; lane 4, after size exclusion chromatography (SEC); lane 5, after ion-exchange chromatography (IEC).

In order to prepare highly purified sIPV, we followed the IPV production process previously published (24) but with modifications (see Fig. S1 in the supplemental material). Stock sIPV was generated from HeLa cells by cotransfecting a cDNA plasmid encoding Sabin poliovirus viral RNA and pREV encoding T7 RNA polymerase. Cultures were monitored until 90% to 95% cytopathic effect (CPE) was observed (25, 26). Viruses were then harvested by freezing and thawing the supernatants and cell mixtures, followed by filtration. This passage 0 (P0) virus stock was used as a working stock to scale up virus production in Vero cells using 3-liter spinner flasks. At 18 h after infection of Vero cells with sIPV P0 stock at a multiplicity of infection (MOI) of 30, the supernatants were collected and freeze-thawed three times. To establish a vaccine production platform mimicking clinical use, we used a multistep purification process that included ultrafiltration, gel filtration, and ion-exchange chromatography. Virus titers were checked at each step to ensure the optimal virus purification and to gain maximal virus recovery (Fig. S1). The purity of virus was confirmed by loading the SDS-treated virion on a PAGE gel, followed by silver staining (Fig. 1). Virus inactivation was carried out by using methanol-free formaldehyde at a final concentration of 0.025% to minimize the capsid protein alteration, and the reaction mixture was then incubated at 37°C. After 14 days, sodium bisulfite was added to neutralize the formaldehyde. The suspension was then dialyzed using a 10-kDa Slide-A-Lyzer cassette against 20 mM sodium phosphate buffer and 25 mM NaCl according to the manufacturer’s instructions.

10.1128/mBio.02287-18.1 FIG S1 Production scheme for lyophilized Sabin inactivated poliovirus. Download FIG S1, PDF file, 0.02 MB. Copyright © 2018 Shin et al. 2018 Shin et al. This content is distributed under the terms of the Creative Commons Attribution 4.0 International license.

### Development of high-throughput SE-HPLC analysis of sIPV.

**FIG 2:**
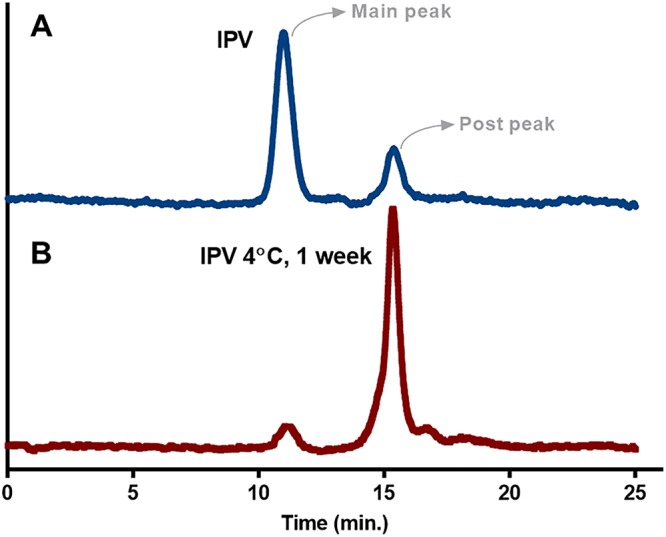
SE-HPLC reliably measures D-antigen stability of sIPV. Poliovirus antigens are divided into D-antigen (D-Ag) and C-antigen (C-Ag); only D-Ag shows major immunogenicity. Size exclusion high-performance liquid chromatography (SE-HPLC) was used as a novel method for determining the antigenicity of sIPV through separation of intact viral particles from disintegrated capsid proteins on the basis of hydrodynamic radius. (A) sIPV chromatogram detecting 336-nm emission from 280-nm excitation through SE-HPLC. (B) SE-HPLC chromatograms of sIPV main peak eluate after 1 week of storage at 4°C in a liquid state.

There are two distinct antigenic forms of PV; infectious virion particles are referred to as D-antigen (D-Ag), and noninfectious empty virion particles are referred to as C-antigen (C-Ag) (22). Due to differences in the antigenic forms of D-Ag and C-Ag, only the D-Ag form of virion particles shows an immunogenic response to viral infection. Moreover, D-Ag can be converted into C-Ag by heating at 56°C; thus, C-Ag is also called H-antigen (H-Ag) (27). The potency of IPVs has been determined by the amount of D-antigens present in the vaccine, typically by enzyme-linked immunosorbent assay (ELISA) (28). Here, size exclusion high-performance liquid chromatography (SE-HPLC) was investigated as a novel method for determining the antigenicity of sIPV through separation of intact viral particles from disintegrated capsid proteins on the basis of hydrodynamic radius. SE-HPLC analysis of sIPV showed one main peak and one postpeak (Fig. 2A), detecting the intrinsic fluorescence of tryptophan residues at excitation lambda/emission lambda (λ_ex_/λ_em_) of 280/336 nm. A preliminary stability test was performed by storing the main peak eluate of sIPV at 4°C for 1 week and subsequently subjecting it to analysis by SE-HPLC. These results revealed that the main peak degraded into the postpeak species and that all degradant species were detected in this method without a loss in total area (Fig. 2B) (Table 1). Dynamic light scattering (DLS) analysis revealed that the main peak observed by SE-HPLC contained a monodispersed particle of 14.6 nm in radius (Table 2) which matched closely with the PV radius of ∼15 nm (1). Eluates of both the main peak and the postpeak from SE-HPLC (Fig. 2A) were collected and measured for the D-AgU by ELISA (29). This also confirmed that only the main peak showed reactivity with the type 1 PV antibody (Fig. S2). These results strongly demonstrated the utility of SE-HPLC as a reliable and efficient method for measuring the stability of sIPV in various lyophilized formulations.

**TABLE 1:** Integration of results from chromatograms shown in Fig. 2

Sabin inactivated poliovirus control sample status	SEC-HPLC peak areas (a.u.)		
	Main peak	Postpeak	Total
Baseline	11.8	3.7	15.5
After 1 wk at 4°C	1.8	14.1	15.9

**TABLE 2:** Dynamic light scattering analysis of main peak

Parameter	Value
Mean radius (nm)	14.6
Pd (%)	8.1
Intensity (%)	100.0
Mass (%)	100.0

10.1128/mBio.02287-18.2 FIG S2 Comparison between D-antigen ELISA and SE-HPLC peaks. Download FIG S2, PDF file, 0.1 MB. Copyright © 2018 Shin et al. 2018 Shin et al. This content is distributed under the terms of the Creative Commons Attribution 4.0 International license.

### Surfactant-based formulation buffer for lyophilization.

A desirable lyophilized formulation should have the following attributes: minimal loss of D-Ag, formation of an elegant cake structure correlating with good product integrity (30), and stability following storage at ambient temperature. A number of traditional stabilizers and lyophilic excipients, including glycine, mannitol, sorbitol, sucrose, and magnesium sulfate, were evaluated, and formulations maintaining D-Ag recovery rates of greater than 80% upon lyophilization were selected for further optimization (Table 3). Following the primary round of screening, the stabilizing effect of surfactants (21) was assessed by agitating the samples for 4 h with and without the addition of 0.01% polysorbate 20, 0.01% polysorbate 80, or 0.1% pluronic F68. While agitation significantly reduced D-antigen levels, the addition of polysorbate 20 or pluronic F68 effectively mitigated D-Ag loss (Fig. S3A and B). Magnesium ion at a concentration of 1 mM was also considered as a stabilizer on the basis of its stabilizing effect on other vaccines, including OPV and IPV (20). Formulation pHs ranging from 6 to 8 were tested, but optimal D-Ag recovery was observed at neutral pH. A histidine buffer used to maintain the neutral pH was combined with mannitol as a bulking agent and sucrose or sorbitol as a stabilizing sugar (the lyophilization conditions are summarized in Table S1 in the supplemental material) (21). This formulation also showed low values of moisture content of 0.77% to 1.30% by Karl Fischer (KF) analysis (Table 4) (17).

**TABLE 3:** List of candidate formulations for sIPV lyophilization and percent recovery of D-antigen following lyophilization^*a*^

Formulation code	Bulking agent/stabilizer	Buffer	Sugar	pH	Surfactant	SE HPLC recovery (%)	ELISA recovery (%)
F1	2.5% glycine	10 mM Tris-HCl	1% sucrose	8	0.1% pluronic F68	89	85
F2	5% mannitol	10 mM histidine	1% sorbitol	7	None	18	19
F3	5% mannitol	10 mM histidine	1% sorbitol	7	0.1% pluronic F68	74	78
F4	5% mannitol	10 mM histidine	1% sorbitol	7	0.5% pluronic F68	91	95
F5	5% mannitol	10 mM histidine	1% sorbitol	7	1.0% pluronic F68	92	91
F6	5% mannitol	10 mM histidine	1% sucrose	7	0.1% pluronic F68	65	88
F7r	5% mannitol	10 mM histidine	1% sucrose	7	0.5% pluronic F68	72	73
F8	5% mannitol	10 mM histidine	1% sorbitol	7	0.01% polysorbate 20	91	90
F9	5% mannitol	10 mM histidine	1% sucrose	7	0.01% polysorbate 20	90	91
F10	5% mannitol	10 mM histidine	1% sorbitol	7	0.1% polysorbate 20	76	81
F11	5% mannitol	10 mM histidine	1% sorbitol	7	0.5% polysorbate 20	77	78
F12	5% mannitol	10 mM histidine	1% sorbitol	7	0.05% polysorbate 20	75	82
F13	5% mannitol	10 mM histidine	1% sucrose	7	0.1% polysorbate 20	80	74
F14	2.5% glycine	10 mM histidine	1% sucrose	7	0.1% pluronic F68	42	76
F15	2.5% glycine	10 mM histidine	1% sorbitol	6	0.1% pluronic F68	59	N.T.
F16	2.5% glycine	10 mM histidine	1% sucrose	6	0.1% pluronic F68	58	N.T.
F17	5% mannitol	10 mM histidine	1% sorbitol	6	0.1% pluronic F68	61	N.T.
F18	2.5% glycine	10 mM histidine	1% sorbitol	6	0.1% pluronic F68	65	N.T.

a MgSO_4_ (1 mM) was used as a stabilizer for all formulations. N.T., not tested.

**TABLE 4:** Moisture content measurements of leading candidates

Formulation code	% moisture content (*t* = 0)
1% water	0.97
F4	0.77
F8	0.97
F9	1.3

10.1128/mBio.02287-18.3 FIG S3 D-antigen recovery. Download FIG S3, PDF file, 0.3 MB. Copyright © 2018 Shin et al. 2018 Shin et al. This content is distributed under the terms of the Creative Commons Attribution 4.0 International license.

10.1128/mBio.02287-18.6 TABLE S1 Parameters for lyophilization cycle. Download Table S1, PDF file, 0.02 MB. Copyright © 2018 Shin et al. 2018 Shin et al. This content is distributed under the terms of the Creative Commons Attribution 4.0 International license.

### Thermostability of lyophilized sIPV and vaccine efficacy *in vivo*.

**FIG 3:**
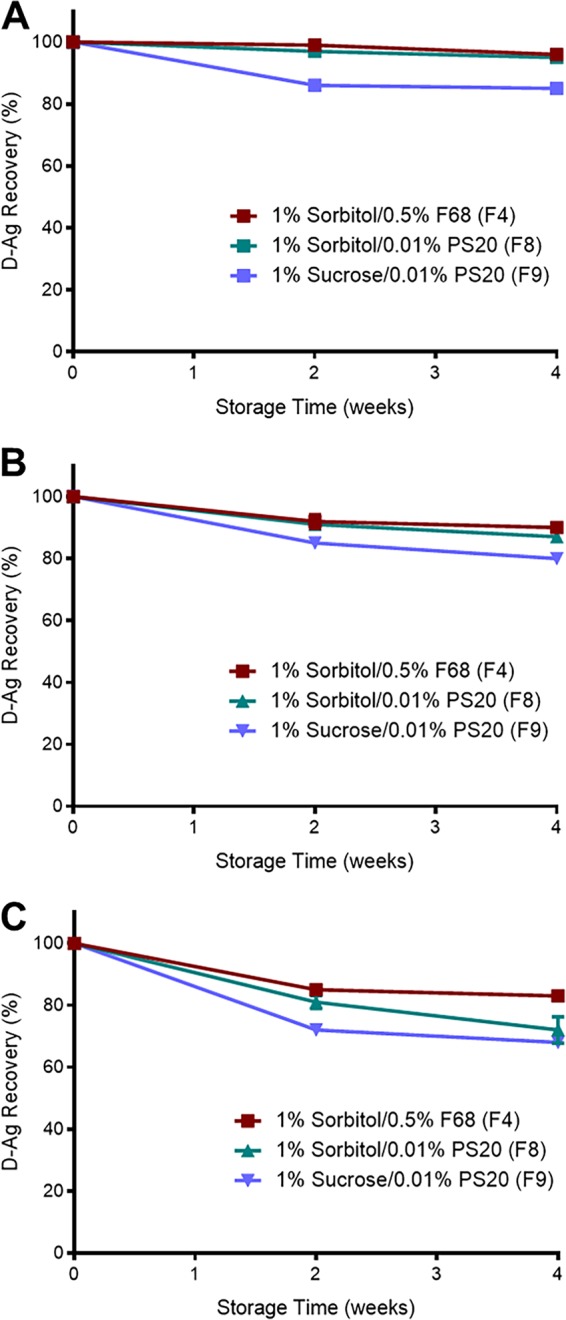
Lyophilized sIPV remains stable at elevated temperatures. To test the thermostability of lyophilized sIPV, lyophilized sIPV from formulations F4, F8, and F9 was incubated at different temperatures and D-Ag recovery was measured using conventional ELISA. (A) D-Ag unit recovery over 4 weeks of incubation at 4°C. (B) D-Ag unit recovery over 4 weeks of incubation at 25°C. (C) D-Ag unit recovery over 4 weeks of incubation in 40°C.

To test the thermostability of the leading lyophilized formulation candidates (formulation codes of F4, F8, and F9 as summarized in Table 3), sIPVs in the formulation were incubated after lyophilization at 4°C (Fig. 3A), 25°C (Fig. 3B), or 40°C (Fig. 3C) for up to 4 weeks. Overall, the leading formulation consisted of 10 mM histidine, 5% mannitol, 1 mM MgSO_4_, 1% sorbitol, and 0.5% pluronic F68 at pH 7 (formulation code F4), with the most efficient results with respect to D-Ag recovery seen at 96%, 90%, and 83% and 4°C, 25°C, and 40°C, respectively. Thus F4 formulation was selected for *in vivo* protective efficacy testing against wild-type (WT) PV infection (31).

**FIG 4:**
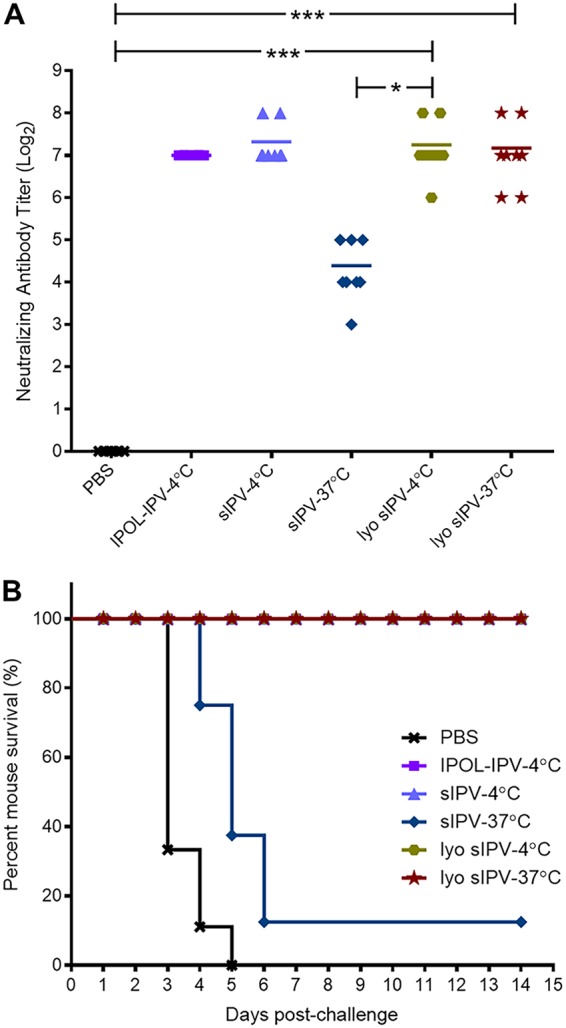
Lyophilized sIPV effectively protects mice against wild-type poliovirus challenge. (A) Mean neutralization antibody titers of vaccination group. Blood of vaccinated cPVR transgenic mice (*n* = 8) treated with commercial IPOL-IPV or sIPV or reconstituted lyophilized (lyo) sIPV incubated at either 4°C or 37°C for 4 weeks was collected at day 21 to measure neutralizing antibody titers against 100 TCID_50_ of Sabin type 1 poliovirus. For the statistical analysis, one-way analysis of variance (ANOVA) (Kruskal-Wallis test) was used. *, P < 0.05; ***, P < 0.001. (B) *In vivo* vaccine efficacy of lyophilized IPV. To investigate the protective efficacy of thermostabilized sIPV *in vivo*, cPVR transgenic mice (*n* = 8) were vaccinated and boosted with commercial IPOL-IPV or sIPV or reconstituted lyophilized sIPV incubated at either 4°C or 37°C for 4 weeks. The mice were then challenged at day 28 with wild-type PV (Mahoney strain) to test virus-induced paralysis. Commercial IPOL-IPV (a trivalent polio vaccine distributed by Sanofi Pasteur) was used as a control.

A total of six groups of poliovirus receptor transgenic (cPVR) mice (*n* = 8) expressing human CD155 for viral entry (32, 33) were vaccinated with 20 D-AgU of sIPV, lyophilized (lyo) sIPV, IPOL-IPV (a trivalent polio vaccine distributed by Sanofi Pasteur), or phosphate-buffered saline (PBS) incubated for 4 weeks at the indicated temperatures. cPVR mice were vaccinated, boosted, challenged, and observed for 2 weeks for signs of paralysis (Fig. S4), using a blind scoring method outlined in the WHO standard operating procedure for OPV neurovirulence testing (34). For the serum neutralizing titers, we first checked day −1 serum and confirmed that no mice had seroconverted, as the neutralization titers were below the detection limit (Fig. S5). We then checked the neutralization titers on days 13 and 21 and found that the neutralization titers measured on day 13 were approximately 2 logs lower than those measured on day 21 (Fig. S5). On the basis of the standardized WHO *in vivo* potency testing of IPV in rodents along with the results of the experiment whose results are shown in Fig. S5, day 21 after vaccination was determined as a time point for the neutralization assay. These assays showed that lyophilized sIPV incubated at 4°C and lyophilized sIPV incubated at 37°C for 4 weeks developed neutralizing antibody titers similar to those seen with nonlyophilized sIPV incubated at 4°C (Fig. 4A). By striking contrast, sIPV incubated at 37°C for 4 weeks showed considerable instability: it developed an approximately 7-fold-reduced neutralizing antibody titer compared to nonlyophilized sIPV incubated at 4°C or lyophilized sIPV incubated at either 4°C or 37°C for 4 weeks (Fig. 4A). Following the boost, cPVR mice were challenged with a 50% paralytic dose (50% PD_50_) of WT Mahoney strain PV. This showed that lyophilized sIPV incubated at 4°C or 37°C for 4 weeks was able to protect mice from paralysis as strongly as commercial IPOL-IPV (Fig. 4B). This unambiguously demonstrated that the lyophilized sIPV remained stable after 4 weeks of incubation at 37°C and induced strong neutralizing antibodies and full protection of poliovirus receptor transgenic mice against *in vivo* challenge with wild-type poliovirus.

10.1128/mBio.02287-18.4 FIG S4 Timeline for *in vivo* survival study of cPVR mice. Download FIG S4, PDF file, 0.1 MB. Copyright © 2018 Shin et al. 2018 Shin et al. This content is distributed under the terms of the Creative Commons Attribution 4.0 International license.

10.1128/mBio.02287-18.5 FIG S5 Determination of optimal time for testing neutralizing antibody titer. Download FIG S5, PDF file, 0.02 MB. Copyright © 2018 Shin et al. 2018 Shin et al. This content is distributed under the terms of the Creative Commons Attribution 4.0 International license.

## DISCUSSION

A majority of human vaccines are temperature sensitive. The dependence of current vaccines on the cold chain, which prevents exposure to ambient temperature and also to freezing (12), presents many obstacles that can lead to failure of vaccination campaigns. As previously reported (35), nearly 1.5 million children lose their lives due to vaccine-preventable diseases. Pharmaceutical Commerce reported that $12.5 billion was spent on cold-chain logistics, of which $9.1 billion was for cold-chain transportation and $3.4 billion was for specialized packaging and instrumentation. Thus, improving methods to generate thermostabilized vaccines can reduce the number of deaths caused by vaccine preventable diseases, and cut down on the expenditure used for cold-chain transport.

This study shows the establishment and optimization of lyophilization conditions to increase the *in vitro* and *in vivo* thermostability and vaccine capacity of sIPV at temperatures up to 40°C for at least one month. The use of SE-HPLC enabled the analysis of various formulations as we were able to distinguish between D-Ag and C-Ag by SE-HPLC, which was later confirmed by ELISA (see Fig. S2 in the supplemental material) and DLS analysis (Table 2) (36). Recent studies have shown the use of SE-HPLC for stability and potency testing assays for human papillomavirus vaccine (37), characterization of influenza vaccine constituents (38), and quality control of vaccines by characterizing the assembly of antigens (39). In agreement with earlier studies using SE-HPLC, this method provides an effective means to screen the vaccine stability and antigen recovery after lyophilization in a high-throughput manner (23) compared to conventional ELISA (29).

In the virus purification step, we used tangential flow filtration (TFF), size exclusion chromatography (SEC), and ion-exchange chromatography (IEC) followed by protein silver staining to ensure the high quality of poliovirus purification. The icosahedral poliovirus nucleocapsid is composed of 60 copies each of four coat proteins (VP1, VP2, VP3, and VP4) (1, 40). As the VP4 protein is very small (∼7 k) and myristoylated, it migrated as broad bands in SDS gel and was not clearly visualized (Fig. 1). However, the calculation of the band density and molecular weight of each VP protein showed that the level of VP4 protein in purified virions was similar to or slightly lower than the levels of the rest of proteins VP1 to VP3. It should be noted that while the D-antigen of poliovirus vaccine carries all four of capsid proteins VP1 to VP4, only the VP1 capsid protein is responsible for the generation of a protective immune response against wild-type virus infection (41).

We screened the optimal lyophilization formulation for minimal D-Ag loss, elegant cake structure reflecting good product integrity, and stability upon storage at ambient temperature. In order to achieve a successful lyophilized poliovirus vaccine for this study, various excipients, including glycine, mannitol, sorbitol, sucrose, and magnesium sulfate, were screened at various concentrations and in various combinations to determine their effectiveness as lyoprotectants during lyophilization and titer recoveries were calculated as normalized results from prelyophilized liquid formulations. Our leading formulation, containing10 mM histidine, 5% mannitol, 1 mM MgSO_4_, 1% sorbitol, and 0.5% pluronic F68 at pH 7, resulted in higher D-Ag recovery following lyophilization and subsequent ambient temperature storage than previous lyophilization of polio vaccine (17). Agitation studies also showed a clear benefit of surfactants such as pluronic F68 or polysorbate 20 for stability under conditions of physical stress (Fig. S3A and B), which is an important factor to be considered during vaccine manufacture (42, 43). Finally, the moisture content of our leading formulation was only 0.77%, which is considerably lower than that seen with previous polio vaccine lyophilization attempts (16). Thus, our leading formulation provides an optimal condition for the stability of sIPV during lyophilization and ambient temperature storage.

Adopting a previously reported vaccination/boosting regimen (44–46), we showed that thermostable lyophilized sIPV incubated at 37°C for 4 weeks induced a potent antipoliovirus immune response in cPVR mice and effectively protected these mice from challenge with the WT PV Mahoney strain. Moreover, the levels of type 1 PV neutralizing antibodies of mice vaccinated with the sIPV F4 formulation were similar to the levels seen with commercial IPOL vaccine. A recent study by Tzeng and colleagues used an injectable microparticle system that releases multiple pulses of antigen over time. Their lead formulation also releases two pulses of antigen 1 month apart, mimicking the vaccination/boosting regimen that is being used in the developing world (47). Although the lyophilization formulation removes any transportation complications due to its long-term stability, we observed that D-AgU level slightly decreased after 4 weeks of incubation at 25°C. Maintaining a consistent D-AgU level for the duration of the month will be paramount for vaccine stockpiles.

The typical maximum amount of time that the vaccine vial is stored at health posts is 3 months. Karp and colleagues (48) have suggested that if the vaccine is stable for more than 2 months, it is possible to remove cold-chain equipment at health posts and stockpile the vaccines. If the vaccine acquires thermostability that lasts more than 12 months, it enables removal of cold-chain equipment at every check point and redesign of the supply chain structure. To address this issue, further optimization experiments are in progress to monitor the vaccine thermostability up to the 3-month and 12-month time points. Therefore, further optimization will hopefully achieve a “truly” temperature-stable polio vaccine. With the endgame of polio eradication in sight, the shift from OPV to IPV will become a necessity to avoid vaccine-borne poliovirus infections. Our study demonstrated that thermostable lyophilized sIPV induces potent antipoliovirus antibodies in cPVR mice and effectively protects mice from WT PV infection. Overall, this novel approach for high-throughput evaluation of antigenicity provides a means to accelerate the process of thermostable vaccine development and to facilitate the availability and efficacy of vaccinations around the world.

## MATERIALS AND METHODS

### Cells and viruses.

Vero cells and HeLa cells were purchased from ATCC and maintained in Dulbecco's modified Eagle's medium (DMEM) (Thermo Fisher Scientific; catalog no. 11965118) containing 10% fetal bovine serum (VWR Life Science Seradigm; catalog no. 1500-500) and 1% penicillin-streptomycin (Thermo Fisher Scientific; catalog no. 15140163). Transient transfections for virus rescue were performed with polyethylenimine (PEI) transfection reagent (Polysciences; catalog no. 23966) according to the manufacturer’s instructions. Scale-up culturing of Vero cells were done in a 3-liter spinner flask (Corning Life Sciences; catalog no. 4502) after attachment of Vero cells to micro-carrier beads (GE Healthcare Life Sciences; catalog no. 17044801). Media were changed every 2 days by removing half of the media and introducing fresh media.

HeLa cells were cotransfected with plasmid pREV encoding T7 RNA polymerase and Sabin strain PV molecular clone (provided by Julie Pfeiffer of the University of Texas Southwestern Medical Center) for 96 h with PEI at a 3:1 ratio. When 90% to 95% CPE was confirmed after 96 h, cells were scraped off and supernatant was collected. The supernatant/cell mixtures were freeze-thawed three times to release the virion from the infected cells and passed through a 0.2-μm-pore-size polyethersulfone (PES) filter (Nalgene; catalog no. 566-0020). The clarified supernatants were loaded at a multiplicity of infection (MOI) of 10 into a dish of Vero cells, and the cells and supernatants were collected after overnight incubation at 32°C. After the supernatants were clarified, the virus suspension was loaded at an MOI of 30 into a suspension of Vero cells in a spinner flask. After overnight incubation at 32°C, the supernatants were collected and freeze-thawed three times.

### Virus purification and inactivation (procedure summarized in Fig. S1 in the supplemental material).

The viral suspension was first clarified by centrifugation and passed through a 0.2-μm-pore-size PES filter. The clarified suspension was concentrated in a laboratory-scale tangential flow filtration system (EMD Millipore; catalog no. XX42LSS11) using a Biomax 100-kDa cartridge (EMD Millipore; catalog no. PXB100C50) at 20 lb/in^2^ input and 10 lb/in^2^ backpressure. Viral concentrate was loaded into a HiPrep Sephacryl S-200 HR column (GE Healthcare Life Sciences; catalog no. 17116601) and run through a 20 mM sodium phosphate buffer (pH 7.0) at 0.5 ml/min on a Duoflow chromatography system (Bio-Rad; catalog no. 7600037). The first 280-nm UV absorption peak was collected and loaded into a 5 ml HiTrap DEAE fast-flow column (GE Healthcare Life Sciences; catalog no. 17-5154-01) using 20 mM sodium phosphate buffer at pH 7.0, and the flowthrough showing a UV 280-nm absorption peak was collected (Fig. 1).

Methanol-free formaldehyde (Thermo Fisher Scientific; catalog no. 28908) (at a final concentration of 0.025%) and M199 media (Sigma-Aldrich; catalog no. M0650) were added into the purified PV suspension. The suspension was then incubated at 37°C for 14 days. The suspension was passed through a 0.2-μm-pore-size PES filter after 1 week to remove any aggregates. After 14 days, sodium bisulfite was added to neutralize the formaldehyde. The suspension was then dialyzed using a 10-kDa Slide-A-Lyzer cassette (Thermo Fisher Scientific; catalog no. 66453) back into 20 mM sodium phosphate buffer and 25 mM NaCl using the manufacturer’s instructions.

### Virus titration and enzyme-linked immunosorbent assay (ELISA) D-antigen unit measurement.

Ten-fold serial dilutions of virus inoculum were absorbed into the well of confluent Vero cells for 90 min in 37°C. After the removal of virus solution, cells were overlaid with 0.75% Avicel–DMEM and incubated in a humidified incubator at 37°C and 5% CO_2_ for 6 days. To visualize the plaques, the cells were fixed by the use of 4% formaldehyde and stained by the use of 0.2% crystal violet solution.

ELISA plates were coated with bovine serum antipoliovirus antibody (National Institute for Biological Standards and Control; catalog no. 234) at a concentration of 1:100. The sIPV was loaded at 2-fold dilutions, and a WHO standard IPV (National Institute for Biological Standards and Control; catalog no. 12/104) was used to produce a standard curve. Mouse monoclonal antibody against type 1 PV (Abcam; catalog no. ab47802) diluted at 1:1,000 was added for detection, and secondary anti-mouse horseradish peroxidase (HRP) antibody (Cell Signaling Technology; catalog no. 7076) diluted at 1:1,000 was loaded. TMB solution (BD Biosciences; catalog no. 555214) was used for quantification on a FilterMax F5 microplate reader (Molecular Devices; F5) after the reaction was stopped using hydrosulfuric acid.

### Size exclusion high-performance liquid chromatography (SE-HPLC).

SE-HPLC was performed with an Agilent 1100 series instrument (Agilent Technologies; catalog no. G1380-90000) equipped with a quaternary pump, a degasser, a temperature controlled autosampler, a UV/Vis diode array detector (DAD), and an Agilent 1200 series fluorescence detector (FLD). A TSKgel G6000PW_XL_ column (7.8 mm by 30 cm) or a TSKgel G3000SW_XL_ column (7.8 mm by 30 cm) purchased from Tosoh Bioscience (King of Prussia, PA) was used. At a flow rate of 0.8 ml/min, 100 µl of sample was injected per analysis. The mobile phase contained 50 mM sodium phosphate–140 mM NaCl at pH 6.7. The FLD was used as the primary detector and was set to acquire data at an excitation wavelength of 280 nm and an emission wavelength of 336 nm. For polio vaccine peak identification, the elution volume and the corresponding retention time of the FLD signal were calculated on the basis of a standard calibration curve. To calculate polio vaccine recovery, the area under the curve of formulations before lyophilization was compared to the area under the curve of formulations after lyophilization.

### Dynamic light scattering (DLS) analysis.

DLS measurements to obtain the mean radius of IPV were performed with a DynaPro plate reader (Wyatt Technology). IPV samples were prepared by dilution of the vaccine 1:1 with phosphate buffer and filtration through a 0.2-µm-pore-size PES filter for DLS analysis. The samples were analyzed at a 25-µl volume in triplicate, and the mean particle radius for the vaccine was calculated via the use of Dynamics software (version 7.1.7).

### Lyophilized cake moisture determination.

The water (or moisture) content in solid lyophilized formulation was determined by coulometric titration using a Mettler C20 Coulometric Titrator (Mettler Toledo; catalog no. 51105510) and an oven. Briefly, each lyophilized sample was brought to room temperature before caps were removed for analysis and subsequently heated in an oven. The moisture released from heating was carried from the oven to the Karl Fischer (KF) titration cell, which contained KF reagent for the reaction. Moisture in the titration cell was continuously titrated until an endpoint was reached. Each sample was measured in duplicate. Once an analysis was complete, results were generated automatically.

### Formulation matrix.

All formulations were prepared at twice the concentration to allow a final 1:1 dilution by volume with the IPV sample. Each stock formulation was prepared by dissolving the stabilizers and bulking agents into a 10 mM histidine buffer containing either polysorbate 20 or poloxamer 188. The adjustment of pH was performed with HCl prior to sterile filtration using a 0.2-µm-pore-size PES membrane. The final IPV-formulation solutions were prepared by mixing equal volumes of IPV sample and stock formulations to generate the formulation matrix for testing. Each mixture was filled into sterilized 2-cm^3^ glass vials (West Pharmaceutical Services) in two replicates and half-capped with sterilized Diakyo 13-mm-diameter serum Flurotec stoppers (West Pharmaceutical Services). All sample preparation and filling process steps were performed under aseptic conditions in a class II biological safety cabinet.

### Lyophilization process design of sIPV.

For this study, all formulations were lyophilized using a conservative cycle designed to generate elegant lyophilized cakes with acceptable moisture content without compromising the vaccine product quality and integrity. The half-capped vials were loaded into a VirTis Genesis 25EL pilot lyophilizer (SP Scientific; catalog no. 100001991) at a shelf temperature of 5°C. Following loading, the vials were slowly cooled until frozen at −50°C, held at the same temperature for 2 h, and subsequently warmed to −15°C at a 0.5°C/min ramp rate. The vials were held at −15°C for 2 h prior to initiation of the primary drying step. The primary drying was performed at a shelf temperature of −15°C and 13.3322 Pa (100 mTorr) chamber pressure for 10 h. The secondary drying step was designed to remove residual water that did not sublimate during the primary drying step; thus, the shelf temperature was increased to 25°C and held for 3 h. After the completion of the lyophilization cycle, the vials were stoppered in a partial vacuum, labeled, and stored at 2 to 8°C before analysis.

### Animal care.

Transgenic poliovirus receptor (cPVR) mice were a gift from Raul Andino (University of California, San Francisco) and were maintained in a University of Southern California (USC) mouse facility according to the university’s regulations for animal care and handling (IACUC).

### Poliovirus challenge.

cPVR mice (6 weeks old; *n* = 8) with confirmed expression of PVR (data not shown) were vaccinated with half of a human dose of IPV (20 DU) via the intraperitoneal (IP) route, and they were boosted with the same dose after 2 weeks. At 14 days following the booster, the mice were injected with WT 1 Mahoney PV at a dose of 50 PD_50_ (50% paralytic dose). Mice were monitored for 2 weeks using a blind paralysis scoring system outlined by the WHO for the mouse neurovirulence test for OPV (the vaccination and challenge procedure is summarized in Fig. S4). If both legs dragged during ambulatory motions, if the legs hung when climbing across a rail, and if the mice were unable to grip the rail, the mice were scored as paralyzed. If the mice maintained a partial ability to move limbs forward and if their legs hung when climbing across a rail but the mice recovered but were still unable to grip the rail, the mice were scored as exhibiting paresis. Paresis mice received a score representing paralysis if they showed signs of paresis for two consecutive days. Mice were euthanized after scoring as paralyzed for a humane endpoint.

### Microneutralization assay.

The neutralization assay followed the WHO standardized protocol for the assay (49, 50) with a little modification. Briefly, mice serum was collected by retro-orbital breeding on day −1 and day 21. The blood was allowed to clot at 4°C for 2 days and then centrifuged at 1,000 × *g* for 30 min at 4°C, and the supernatant was collected. After heat inactivation, sera were diluted in DMEM in a 2-fold serial dilution and an equal volume of 100 50% tissue culture infective doses (TCID_50_) of WT Mahoney PV was added and incubated for 3 h in 37°C. These mixtures were then infected in Vero cells (about 80% to 90% confluent) plated in a 96-well plate in 4°C for 18 h, washed with PBS, and further incubated for 5 days in DMEM. Neutralization antibody titers were calculated from dilutions that corresponded to a 50% reduction of virus infection compared to control.
