# Supplementary figures and images for: Development of Thermostable Lyophilized Sabin Inactivated Poliovirus Vaccine
Source: mBio. 2018 Nov 27;9(6):e02287-18. doi: 10.1128/mBio.02287-18 (PMC6282204; doi:10.1128/mBio.02287-18)

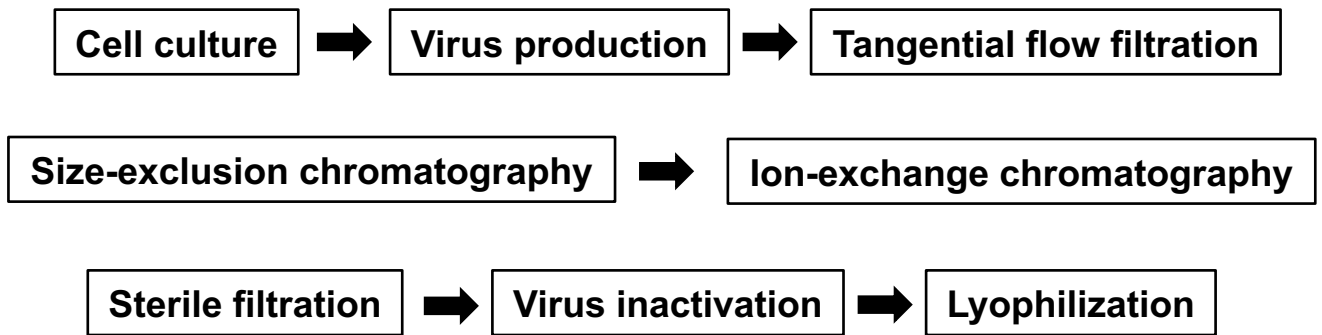

**Fig. S1.** Production Scheme of lyophilized Sabin inactivated Poliovirus

Supplement: FIG S1 [file mbo006184192sf1.pdf]

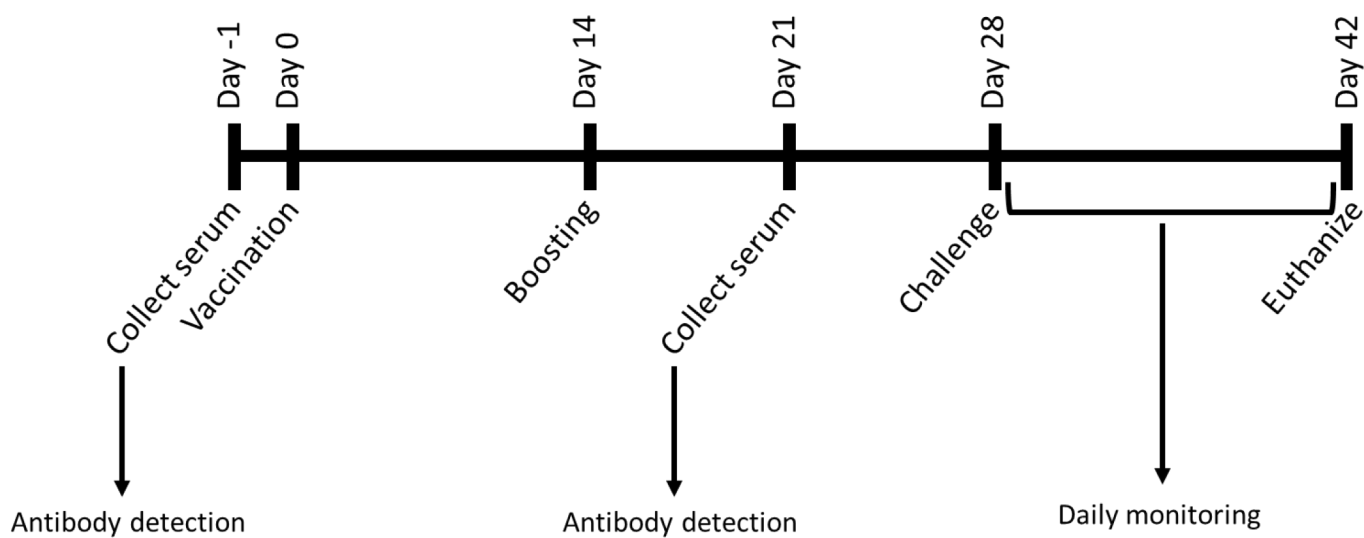

**Fig. S4.** Timeline for *in vivo* survival study of cPVR mice.

Supplement: FIG S4 [file mbo006184192sf4.pdf]
