# Supplementary material for: Development of Thermostable Lyophilized Sabin Inactivated Poliovirus Vaccine
Source: mBio. 2018 Nov 27;9(6):e02287-18. doi: 10.1128/mBio.02287-18 (PMC6282204; doi:10.1128/mBio.02287-18)
Supplement: FIG S2 [file mbo006184192sf2.pdf]

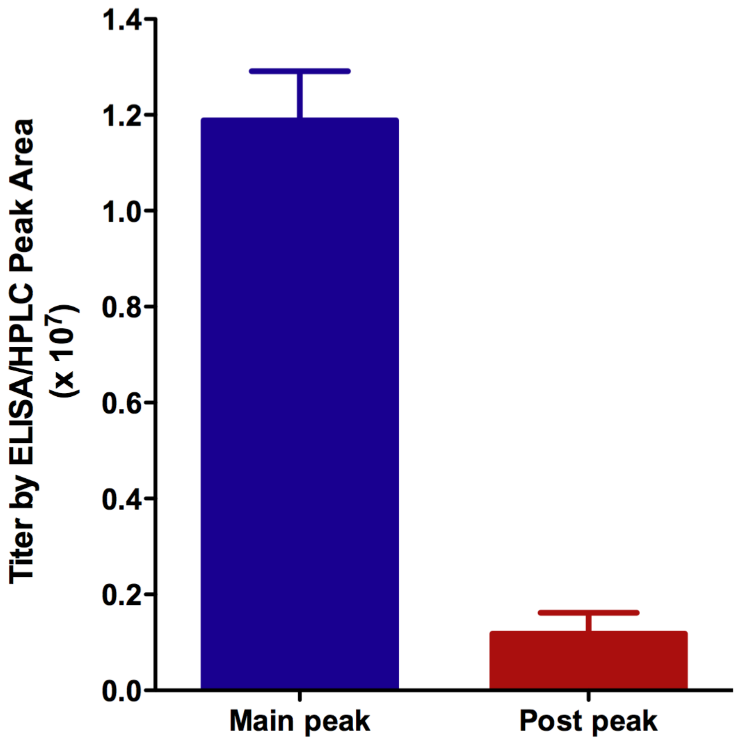

Fig. S2. Comparison between D-Antigen ELISA and SE-HPLC peaks. The relative potency of the main peak and post peak was measured by dividing the ELISA titer of each peak by the area under the UV peaks. Peak areas are shown in supplementary table 2.
