# Supplementary material for: Development of Thermostable Lyophilized Sabin Inactivated Poliovirus Vaccine
Source: mBio. 2018 Nov 27;9(6):e02287-18. doi: 10.1128/mBio.02287-18 (PMC6282204; doi:10.1128/mBio.02287-18)
Supplement: FIG S3 [file mbo006184192sf3.pdf]

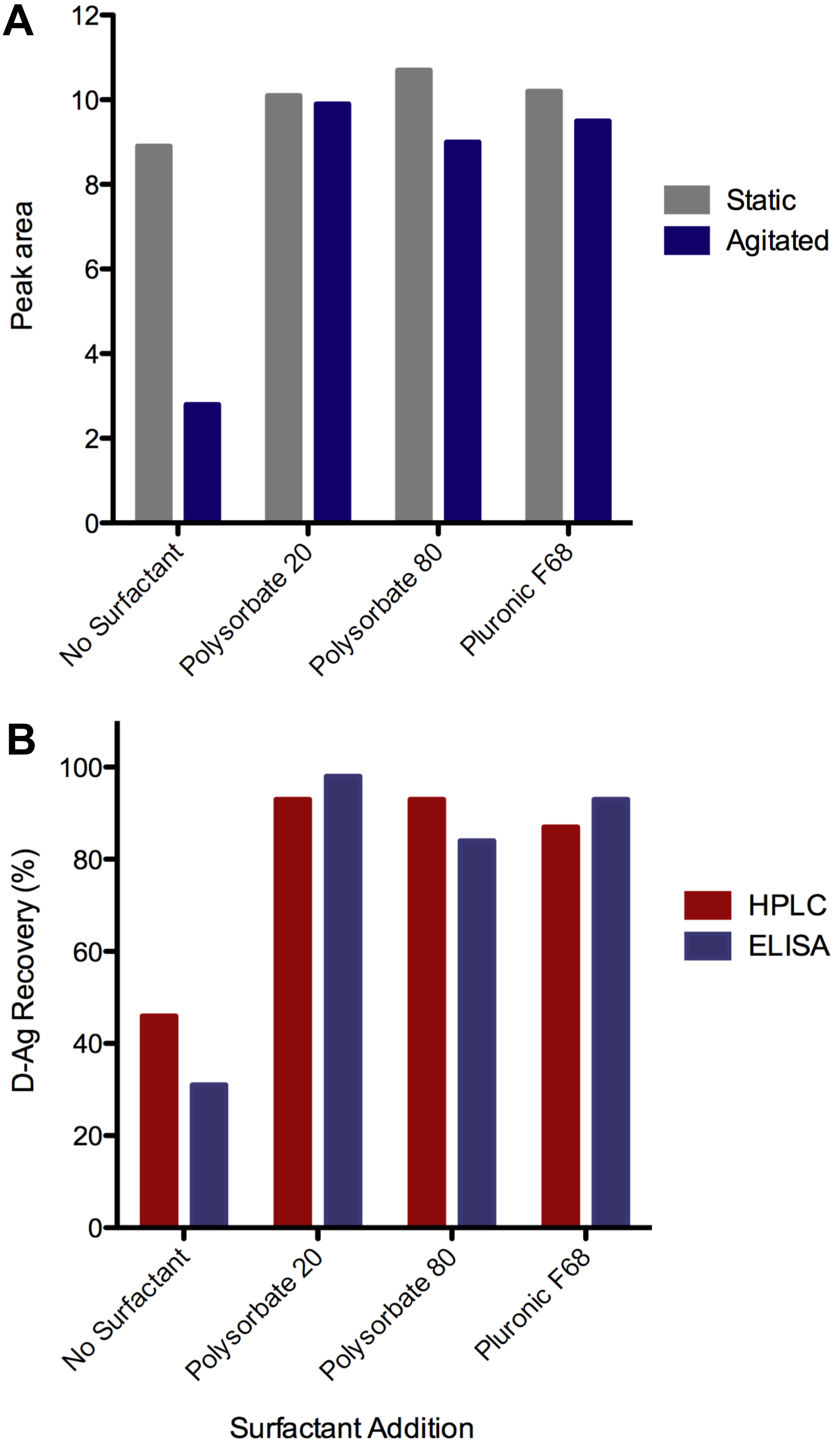

Fig. S3. D-antigen recovery

A. D-antigen area from SE-HPLC of static and agitated samples.

B. Percent D-antigen unit recovery after agitation with different surfactants.
