# Supplementary material for: Development of Thermostable Lyophilized Sabin Inactivated Poliovirus Vaccine
Source: mBio. 2018 Nov 27;9(6):e02287-18. doi: 10.1128/mBio.02287-18 (PMC6282204; doi:10.1128/mBio.02287-18)
Supplement: TABLE S1 [file mbo006184192st1.pdf]

Table S1. Parameters for lyophilization cycle

Step	Temperature	Time (min)	Ramp Rate (°C/min)	Chamber Pressure (mT)
Loading	5°C	N/A	N/A*	N/A
Freezing	5°C to -50°C	110 min	0.5°C/min	N/A
	-50°C	120 min	N/A	N/A
	-50°C to -15°C	70 min	0.5°C/min	N/A
	-15°C	120 min	N/A	N/A
Primary Drying	-15°C	700 min	N/A	100 mT
Secondary Drying	-30°C to 25°C	80 min	0.5°C/min	100 mT
	25°C	120 min	N/A	100 mT

* Not applicable
