# Supplementary material for: Development of Thermostable Lyophilized Sabin Inactivated Poliovirus Vaccine
Source: mBio. 2018 Nov 27;9(6):e02287-18. doi: 10.1128/mBio.02287-18 (PMC6282204; doi:10.1128/mBio.02287-18)
Supplement: FIG S5 [file mbo006184192sf5.pdf]

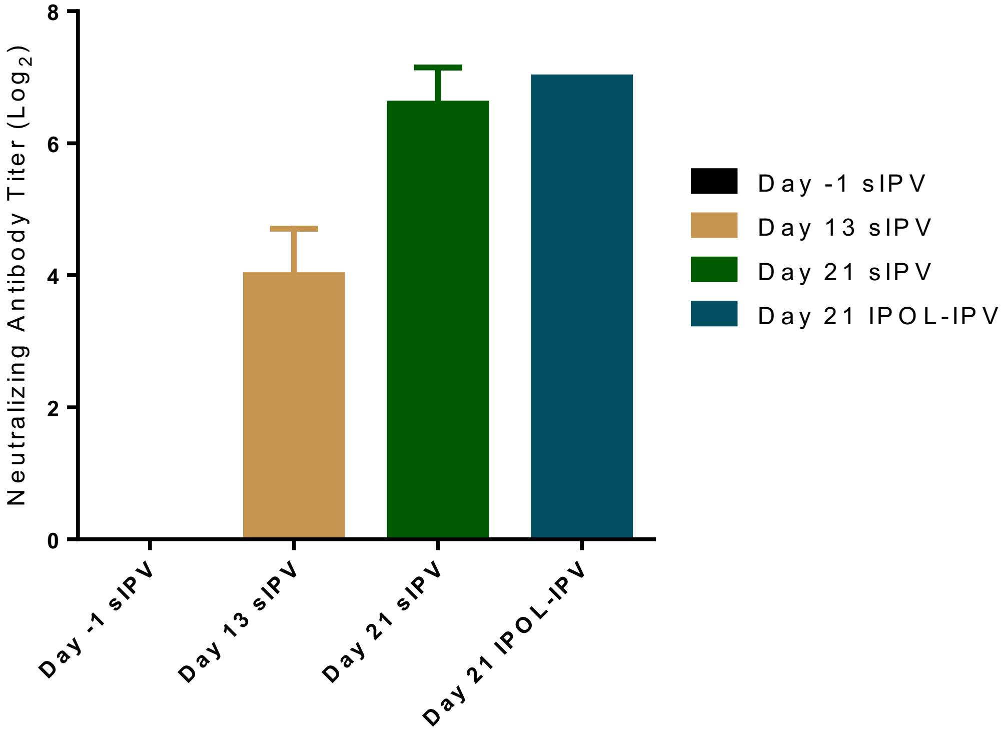

Fig. S5. Determination of optimal time for testing neutralizing antibody titer. Blood of vaccinated cPVR transgenic mice (n=8) sIPV was collected at days 13 and 21 to measure neutralizing antibody titers against 100TCID$_{50}$ of Sabin type 1 poliovirus. Commercial IPOL-IPV was included as a control.
